# Health facilities readiness to provide primary cardiometabolic healthcare in Burkina Faso, 2012-2018

**DOI:** 10.1093/eurpub/ckac129.651

**Published:** 2022-10-25

**Authors:** K Cissé, F Kirakoya

**Affiliations:** 1 Biomedical and Public Health, Research Institute of Health Sciences, Ouagadougou, Burkina Faso; 2 Ecole de Santé Publique, Université Libre de Bruxelles, Brussels, Belgium

## Abstract

**Background:**

Cardiovascular diseases (CVD) and diabetes, referred to as “cardiometabolic diseases” (CMD), are a growing health issue in developing countries like Burkina Faso. As the first contact point with the national health system, primary health care must play a crucial role in CMD prevention and control. This study aimed to analyse the primary health care (PHC) system readiness for CMD prevention and treatment in Burkina Faso from 2012 to 2018.

**Methods:**

We performed repeated cross-sectional data analysis from health facility-based surveys, conducted in 2012, 2014, 2016, and 2018. These surveys were conducted using the World Health Organisation (WHO) Service Availability and Readiness Assessment (SARA) tool. The readiness of PHC for CMD was defined according to the SARA manual.

**Results:**

A total of 586 healthcare facilities were included in 2012, 659 in 2014, 567 in 2016, and 653 in 2018. Between 2012 and 2018, the percentage of healthcare facilities providing CMD specific care significantly increased (66.2% to 92.0% for CVD and 39.4% to 46.6% for diabetes). However, the mean readiness index of the PHC system to manage CVD decreased from 26.0% to 21.6% (p for trend<0.001). For diabetes, the overall readiness index increased significantly (from 34.2% to 37.5%, p = 0.005). The readiness index of PHC for CVD significantly decreased in all health regions particularly in the Sahel region (from 31.7% to 20.8%, p < 0.001). While, for diabetes, it increased in all the health regions excepted the Centre-Sud region (from 37.8% to 32.2%, p < 0.001).

**Conclusions:**

There is a low level of preparedness of PHC system to provide CMD in Burkina Faso. Although improvements for diabetes, this is not enough (80% availability of affordable basic technologies and essential medicines recommended by the WHO). Strengthening of the primary healthcare system considering the geographical disparities is urgently required for early detection and management of CMD.

**Key messages:**

• There is a low level of readiness of PHC system to provide cardiometabolic healthcare in Burkina Faso.

• Public health policy makers must pay more attention to strengthening of the primary healthcare system to address the rising burden of CMD.

